# Decision Making for Active Surveillance in Vestibular Schwannoma

**DOI:** 10.1097/ONO.0000000000000022

**Published:** 2022-12-22

**Authors:** Harrison Smith, Ahmad Odeh, Dorina Kallogjeri, Jay F. Piccirillo

**Affiliations:** 1 Department of Otolaryngology-Head and Neck Surgery, Mount Sinai/New York Eye and Ear, New York, NY; 2 Saint Louis University School of Medicine, St. Louis, Missouri; 3 Department of Otolaryngology, Head and Neck Surgery, Washington University in St. Lous, Missouri.

## Abstract

**Objective:**

To describe the experiences of patients who elected for the active surveillance treatment option for their vestibular schwannoma (VS).

**Study Design:**

Twenty-two patients participated in semistructured patient interviews.

**Setting:**

Interviews were conducted between March and April 2021 via telephone with audio recordings and notes taken during each interview.

**Patients:**

Adults diagnosed with a VS and at any point after their diagnosis underwent a period of active surveillance were recruited based on the diagnosis made by MRI. Patients were excluded if they chose to undergo treatment immediately, had a diagnosis of neurofibromatosis type 2, or if they had a confirmed alternative diagnosis.

**Intervention:**

This intervention was a qualitative interview to assess patient experiences with their VS treatment decision.

**Main Outcome Measures:**

Identifying abstract categories that represent many of the stories told by the participants that produces a theory grounded in the data with explanatory power.

**Results:**

Factors that influenced patients’ treatment decisions were perceived physician bias, selfeducation, and personal accounts of VS patients through support groups, and side effects/complications of the various treatment options.

**Conclusion:**

Patients who opted for active surveillance as a treatment option reported high satisfaction with their decision and greater confidence in future treatments that would be necessary based on tumor growth. Future work should be done to increase shared decision making between the physician and patient to arrive at a treatment plan that aligns with their goals of care as well as potentially reducing overtreatment of VS.

The management of vestibular schwannoma (VS) has always presented unique challenges to patients and physicians. With the increased incidence of these benign tumors over the last 40 years, there is even more importance on selecting treatment pathways that optimize patient outcomes (1–3). This increase in incidence is likely a result of increased access to healthcare and advances in imaging technology and has also resulted in a trend toward patients being diagnosed with increasingly small tumors compared to years prior. The typical management pathways for VS include surgical removal, radiation therapy, and active surveillance. Each of these options offers a unique set of risks and benefits that are tolerated at different levels. Surgical intervention places patients at risk of facial paralysis, hearing loss, and intraoperative complications with the benefit of definitive tumor control (4–6). Radiation therapy largely avoids risks of facial paralysis and operative complications at the cost of exposing patients to a long-term hearing loss and providing less robust tumor control (7,8). Active surveillance lacks the immediate risks that accompany active treatments without providing any intervention for tumor control, instead relying on the tumor’s natural progression to remain indolent. With tumor diagnosis occurring at smaller tumor sizes, the focus of treatment turns to a risk benefit conversation with patients that attempts to align the patient’s goals for their care with the appropriate management pathway (9). Among patients who decide to undergo active surveillance, some live years without any significant tumor growth (10–13). Even for patients who experience tumor growth, this growth is not always be sustained (14). Under the correct circumstances, long-term active surveillance may be the optimal treatment strategy for a subset of VS patients. Given the trend of increased detection and acknowledgement of the successes of conservative management, it is important to recognize the likelihood that this benign tumor is subject to unnecessary treatment (15). In consideration of this, we looked to explore the decision-making process of people who elected to undergo active observation after their VS diagnosis. Our aim was to describe the experience of patients making this decision to identify common experiences as well as opportunities to assist newly diagnosed patients in their own decision-making process.

## MATERIALS AND METHODS

### Inclusion Criteria

Inclusion criteria included adults diagnosed with a sporadic VS who at any point after their diagnosis underwent a period of active surveillance. Given that many of these patients would not have pathologic confirmation of their diagnosis, we relied on the diagnosis made on MRI.

### Exclusion Criteria

Patients were excluded if they chose to undergo treatment immediately, if they had a diagnosis of neurofibromatosis type 2, or if they had a confirmed alternative diagnosis.

### Recruitment

**FIG. 1.**
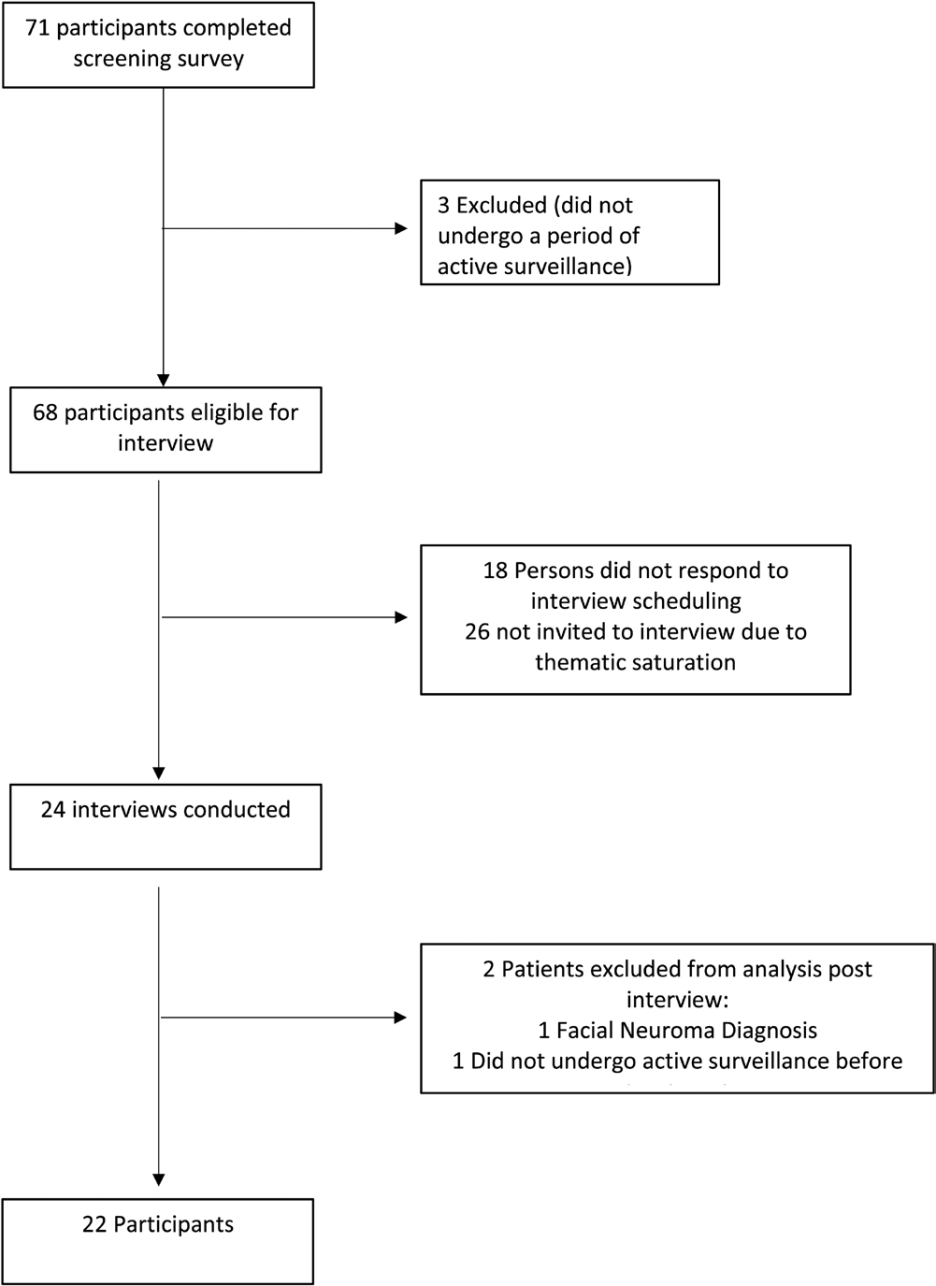
Flowchart of patient recruitment.

Patients were recruited in partnership with the Acoustic Neuroma Association (ANA). Members were made aware of the study by email newsletter and provided with study recruitment flier. Additionally, study information and a recruitment flier were published on the ANA website, allowing for participation of any visitor to their webpage. A flowchart of patient recruitment is outlined in Figure 1.

### Study Design

Twenty-two participants completed 1 semistructured interview each. Interview guide for semistructured interviews is available from the authors. Interviews were conducted by author H.J.S. between March and April 2021 via telephone. Audio recordings and field notes were taken during each interview. Audio recordings were transcribed verbatim.

### Analysis

Analysis of transcribed interviews was done utilizing the grounded theory methodology. Transcripts were initially coded using a structured code book. Throughout the interview process, these codes were revisited and additional codes added based on the content of subsequent interviews. Whenever a new code was added to the code book, previous interviews were reviewed for identification of the new code. After the completion of initial coding, coding of sections was reviewed with the research group. Once consensus on coding was reached an iterative process of identifying categories that conceptually linked concepts identified by coding was completed. The goal of this process was to identify abstract categories that represent many of the stories told by the participants that produces a theory grounded in the data with explanatory power.

## RESULTS

Of the 22 participants in this study, 12 eventually received treatment for their VS while 10 are still undergoing active observation. The median length of time in active surveillance for the participants who received treatment was shorter than patients who are still undergoing active observation. The median reported tumor size was 1 cm in maximum diameter. The largest tumor reported was 2.3 cm with the smallest was 0.1 cm. All participants identified as white. Participants represented a wide range of geographic locations within the United States (Table 1).

### The Experience of Active Surveillance in Vestibular Schwannoma

**FIG. 2.**
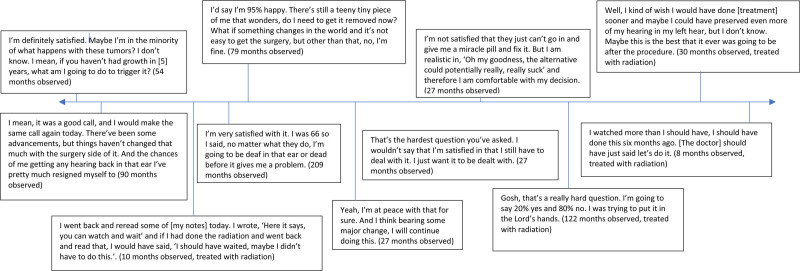
The major domains of experience of the participants who elected to undergo a period of active observation.

The major domains of experience of the 22 participants who elected to undergo a period of active observation is shown in Figure 2. These domains are organized based on a chronologic timeline of the decision-making process. Within each domain, associated themes are listed accompanied by excerpts that highlight the components of this theme. After their diagnosis, the participants began a period of information seeking. Using this information, participants conducted a harm-benefit analysis to arrive at their decision. The participants then reflected on their decision to provide advice to other patients and remark on their satisfaction.

To learn more about their diagnosis and available treatments, participants sought out information from a variety of sources. Common to all the participants was seeking advice from their physician and many participants found value in seeking the opinion of many different physicians. Often these discussions led to a treatment recommendation, but in some cases the physician left it to the participant’s discretion. Occasionally, participants felt that a recommendation was biased due to the physician’s training, experience, or ongoing research. Additionally, participants relied on online resources and personal accounts from other VS patients. The ANA website was a common source that people used to read articles on treatment options and attend educational webinars. Many also used it to communicate with other VS patients in person, over the phone, or via online forums. In some cases, communication with other VS patients was facilitated by the participant’s physician. Few participants reported having no interest in hearing the experiences of other VS patients.

After assimilating this information, many described arriving at their decision to undergo active observation after conducting a harm-benefit analysis of the available options. There were no unifying criteria that each participant used in this analysis but the major considerations participants made involved the potential of side effects from the treatment, the severity of their symptoms, their tumor size and rate of growth, and other factors in their life. Participants concerned with side effect from treatment highlighted the risk of facial paralysis, long-term headaches, deafness, and balance disturbance. Participants with a small, indolent tumor, or mild symptoms frequently questioned the benefit that treatment would provide them. Many felt that the severity of their diagnosis did not justify pursuing treatment when considering the risks. For others, the demands of caretaking for children and parents took precedent over a condition that they felt was not a threat to their own wellbeing. Importantly, many used their tumor growth as a key factor in making their decision. In instances where participants were ultimately treated, it was often detected tumor growth on subsequent scans that led to a change in treatment strategy.

**FIG. 3.**
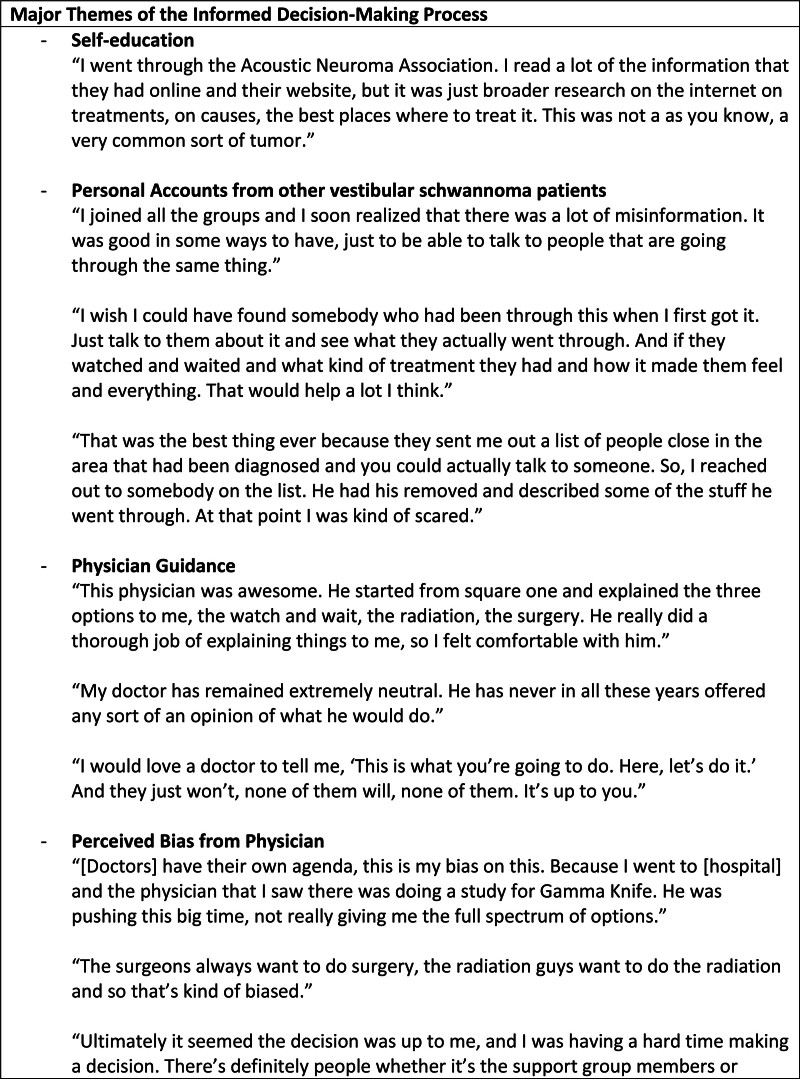
Major themes of the informed decision-making process.

Upon reflection, most of the participants were satisfied with their decision and recommended for newly diagnosed patients to do the same (Figure 3). This held true even among participants who did undergo treatment. The participants emphasized the benefit of having time to learn more about their diagnosis as well as the opportunity to discuss the experience of other persons. Many cited the challenge of fully grasping the reality of their diagnosis in the immediate period following the discovery of their VS. When participants reported dissatisfaction, it was often because they felt earlier intervention may have preserved their hearing. Of those treated, many reported the benefit of having more time to learn about their diagnosis before making a decision as well as the peace of mind knowing that their tumor was actively growing before they pursued intervention. Those still in active surveillance reported satisfaction as well, particularly those who had maintained stable tumor size and symptoms over the years.

## DISCUSSION

Newly diagnosed patients with VS potentially stand to benefit from the conservative approach chosen by the participants in our study. Given the increased rate of detection of these tumors, there is certainly a portion of patients who in years past may have otherwise lived their lives without ever knowing of their tumor. Due to its classification as a brain tumor and limited public awareness, many patients experience a sense of urgency and anxiety after diagnosis (16). These circumstances set the stage for the over treatment of a benign condition. Not only can unnecessary treatment have long-lasting impacts on patient’s quality of life but also it may have implications on healthcare waste and spending (17–19).

Despite this difficult diagnosis, the participants in our study chose to undergo active surveillance after consolidating information from a variety of sources. In pursuing information online, from their physicians, as well as from other people with VS, our participants each underwent an individualized harm-benefit analysis that led them to their decision. Given the main benefit that treatment for VS can provide is tumor control and, in some cases, hearing preservation, it is understandable that the perceived risks of treatment would take precedent. In many cases, the appreciation for the risks involved with treatment was amplified after hearing stories from other VS patients. This was exemplified in discussions around facial paralysis as the visual stimuli in combination with emotional connection to a person’s story can be especially provocative.

The value of connecting newly diagnosed patients with others who have VS is substantial but comes with it its own risks. The communication with others previously diagnosed with VS can be helpful in several ways: it can draw attention to questions that a newly diagnosed patient had not previously considered, it can allow patients to better understand the logistics behind various treatment options, and it can take the information about VS from an impersonal webpage or pamphlet to a real human experience. That said, people who seek out these connections are at risk of receiving misinformation about VS as well as a prospective that is not completely holistic. In this sense, it may be beneficial for physicians to acknowledge the reality that many patients do want to solicit advice and experiences from other VS patients and facilitate a more balance prospective from their own patients.

Although persons with VS may have preconceived notions about the treatment options for VS, physicians are also prone to their own bias’. Given the duty physicians have to the wellbeing of their patients, it is not surprising that physicians may promote treatment options that they feel are best suited for their patients. In some circumstances, this was met by skepticism by patients. Generally, this skepticism stemmed from the notion that physicians are drivers of the business of healthcare. Other instances referred to other potential conflicts of interests, including ongoing research, and previous training. This perception is the reality for many patients and emphasizes the importance of building a trusting relationship through education and transparency.

There remains a need for a centralized resource that addresses common questions about VS treatment in a complete and concise manner. A diagnosis of a VS comes with a lot of uncertainty and an overwhelming amount of information. To expect patients to be able to seek out and understand this information independently is unrealistic. Although there are outstanding resources that currently exist, such as the ANA, these are often sought out independently after the diagnosis. To facilitate a better understanding of the options available as well as engage a shared decision-making process between patient and physician, a decision aid, such as a decision grid could provide great benefit.

## CONCLUSION

Participants undergoing active surveillance for VS reported high satisfaction with their decision. Participants reported the benefits of having time to better educate themselves, whether it was through additional physician consultation, discussion with other VS patients, or independent research using online resources were core to their satisfaction. Additionally, having information of further tumor growth allowed those who did undergo treatment to feel confident that electing for treatment was not an unnecessary escalation of care. Further work to assist patients and their physicians in facilitating these conversations surrounding treatment will benefit VS patients to arrive at a treatment plan that aligns with their goals of care as well as potentially reduce overtreatment of VS.

**Table 1.** Participant demographic and treatment information

Sex (%)	
Female	14 (66.7%)
Race/Ethnicity	
White	21 (100%)
Age at diagnosis, years (min-max)	55 (37–75)
Size at diagnosis (min-max)	1 cm (0.2–2.3)
Received treatment	10 (48%)
Length of observation, mo (min-max)	
Not treated	79 (6–209)
Treated	22 (3–122)
Location	
Massachusetts	2
Florida	4
Georgia	3
Missouri	2
Maryland	2
Pennsylvania	1
New York	1
New Jersey	1
California	2
Washington	1
Kentucky	1
Puerto Rico	1
Hawaii	1

## FUNDING SOURCES

Dr. Smith received support from the National Institute of Deafness and Other Communication Disorders within the National Institutes of Health, through the “Development of Clinician/Researchers in Academic ENT” training grant, award number T32DC000022. The content is solely the responsibility of the authors and does not necessarily represent the official views of the National Institutes of Health.”

## CONFLICT OF INTEREST

None declared.

## DATA AVAILABILITY

The datasets generated during and/or analyzed during the current study are not available.
